# Holmium laser lithotripsy improves the rate of successful transcystic laparoscopic common bile duct exploration

**DOI:** 10.1007/s00423-019-01845-3

**Published:** 2019-12-11

**Authors:** Timothy Jones, Jasim Al Musawi, Lalin Navaratne, Alberto Martinez-Isla

**Affiliations:** 1 Department of Upper GI Surgery, Northwick Park and St Mark’s Hospitals, London North West University Healthcare NHS Trust, Watford Road, Harrow, HA1 3UJ UK; 2 Department of Surgery and Cancer, Imperial College London, South Kensington Campus, London, SW7 2AZ UK

**Keywords:** Holmium, Laser, Lithotripsy, Choledocholithiasis, Laparoscopic, Common bile duct

## Abstract

**Purpose:**

Transcystic laparoscopic common bile duct exploration (LCBDE) seems safer than transductal LCBDE and is associated with fewer biliary complications. It has traditionally been limited to smaller bile duct stones however. This study aimed to assess the ability of laser-assisted bile duct exploration by laparoendoscopy (LABEL) to increase the rate of successful transcystic LCBDE in patients with bile duct stones at the time of laparoscopic cholecystectomy.

**Methods:**

Patients undergoing LCBDE between 2014 and 2018 were retrospectively analysed. Baseline demographic and medical characteristics were recorded, as well as intra-operative findings and post-procedure outcomes. Standard LCBDE via the transcystic route was initially attempted in all patients, and LABEL was only utilised if there was failure to achieve transcystic duct clearance. The transductal route was utilised for failed transcystic extraction.

**Results:**

One hundred and seventy-nine consecutive patients underwent LCBDE; 119 (66.5%) underwent unaided transcystic extraction, 29 (16.2%) required LABEL to achieve transcystic extraction and 31 (17.3%) failed transcystic extraction (despite the use of LABEL in 7 of these cases) and hence required conversion to transductal LCBDE. As such, LABEL could be considered to increase the rate of successful transcystic extraction from 66.5% (119/179) to 82.7% (148/179). Patients requiring LABEL were however more likely to experience major complications (CD III–IV 5.6% vs 0.7%, p = 0.042) although none were specifically attributable to the laser intra-operatively.

**Conclusions:**

LABEL is an effective adjunct to LCBDE that improves the rate of successful transcystic extraction.

## Introduction

Common bile duct (CBD) stones are reported in up to 15% of patients with symptomatic gallstones requiring laparoscopic cholecystectomy (LC) [1–3]. Only a minority of these stones pass spontaneously, and therefore the majority of patients require invasive intervention [4].

Current UK guidelines suggest that LCBDE at the time of LC as a single-staged procedure is the preferred technique for the management of gallstones with concomitant bile duct stones [5]. Although more technically challenging, transcystic (TC) LCBDE is associated with less morbidity when compared to transductal (TD) LCBDE via choledochotomy [6]. Feng et al. performed a meta-analysis of almost 3000 patients and demonstrated that TC LCBDE had a lower biliary complication rate and shorter hospital stay compared to TD LCBDE [7]. They key issues with choledochotomy and TD LCBDE relate to loss of CBD integrity, resulting in higher incidences of bile leak and haemobilia/blood loss, the need for T-tube insertion (with corresponding complications such as electrolyte imbalance) and the need for further invasive intervention [8].

TC LCBDE has traditionally been limited to smaller CBD stones that are able to pass into the duodenum after flushing or undergo extraction via the cystic duct using standard retrieval techniques. Strömberg et al. suggested that stones exceeding 5 mm in size have a much lower likelihood of TC extraction and are susceptible to impaction [9]. As such, a variety of methods have been reported for improving extraction rate via the TC route including robotic techniques [10], micropercutaneous nephrolithotomy [11], electrohydraulic lithotripsy (EHL) [8, 12] and, more recently, holmium laser lithotripsy [13]. The use of holmium laser treatment is already well established in urological surgery, including its use in ureteric stones, enucleation of the prostate, strictures and bladder tumours [14]. Varban and colleagues were the first group to publish a case series on the use of holmium laser lithotripsy for CBD stones [13]. In 2017, our group published our initial experience of laser-assisted bile duct exploration by laparoendoscopy (LABEL) for the management of complex choledocholithiasis, thereby improving surgical outcomes and reducing technical failure [15]. Further to our report, other groups have also been able to demonstrate safety and efficacy of laser lithotripsy when used as an adjunct to TC LCBDE for large and/or impacted CBD stones [16, 17].

The aim of this present study was to assess the ability of LABEL to increase the success rate of TC LCBDE, whilst also evaluating its safety profile in a contemporary cohort of patients.

## Materials and methods

### Patient inclusion

A retrospective review of a prospectively collected database of 179 consecutive patients who underwent LCBDE at a single centre between February 2014 and December 2018 was performed. All operations were performed or supervised by the senior surgeon (AI). After review of the medical records, investigation results and operative notes, all patients were assigned into two groups based on whether or not LABEL was utilised. All patients were assessed with pre-operative liver function tests (LFTs) and abdominal imaging. Patients in our institution routinely undergo pre-operative ultrasound and then intra-operative cholangiography (IOC) to identify CBD stone burden and position. In patients with very high bilirubin levels or where there is clinical suspicion of another pathology, such as a biliary malignancy, patients undergo cross-sectional imaging, usually magnetic resonance cholangiopancreatography (MRCP). In our centre, patients with acute cholecystitis and CBD stones routinely undergo LCBDE, with endoscopic retrograde cholangiopancreatography (ERCP) being reserved for high-risk surgical patients, patients diagnosed with acute cholangitis and those patients who have had cholecystitis for greater than one week, in line with international guidelines [5].

Data collected included pre-operative demographic information, medical co-morbidity, pre-operative investigations, intra-operative findings and post-operative outcomes. Clinical presentation was classified into four groups: dilated CBD, deranged LFTs, jaundice and pancreatitis. Patients with bilirubin more than two times the upper limit of normal were classified as ‘jaundiced’ irrespective of the liver enzymes (alanine aminotransferase (ALT) and alkaline phosphatase (ALP)) or diameter of the CBD. Those patients with abnormal liver enzymes but bilirubin within the normal range or less than two times the upper limit of normal were classified as ‘deranged LFTs’ irrespective of CBD diameter, whereas patients with intra- or extra-hepatic duct dilatation on pre-operative imaging and normal bilirubin and liver enzymes were classified as ‘dilated CBD’. Outcomes of this study were stone clearance rates, TC exploration rate, conversion to open surgery, post-operative complications (Clavien-Dindo classification) and length of post-operative hospital stay. Length of post-operative hospital stay was chosen instead of total length of hospital stay because patients with acute cholecystitis, obstructive jaundice or pancreatitis were often admitted under the emergency surgery service and remained inpatients until their operation could be scheduled on to the next dedicated biliary operating list.

### Operative technique and indications for LABEL

Patients were formally consented in accordance with both local and international guidelines, including the use of LABEL, failure of stone extraction and further endoscopic or surgical procedures. At our institution, it is routine practice to attempt LCBDE with a choledochoscope via the TC route in the first instance, utilising either water flushes or the Dormia basket for retrograde retrieval. The use of LABEL was indicated when standard Dormia basket retrieval had failed via the TC route. Reasons for failure of ‘standard’ stone extraction included stone impaction within the CBD or stones too large to be retrieved via a smaller cystic duct. Cases initially failing TC extraction either underwent LABEL or were directly converted to TD LCBDE (e.g., if rapid completion was necessary or if the operator judged the TC route to be impossible). Cases that failed TC LABEL were also converted to TD LCBDE. A laser-amenable theatre with trained staff and appropriate safety provisions in place, including a laser checklist, was used for all LABEL cases. Utilisation of the holmium laser was conducted in accordance with our previously described technique [15]. In brief, the fibre-optic holmium laser with a calibre range of 200–365 μm was introduced through the working channel of the choledochoscope (ScopeSafe^TM^, Optical Integrity, Florida, USA). Lithotripsy was achieved by aiming a light diode at the stone and activating the laser. Initial laser energy was set to 0.5 J (with a frequency of 20 Hz) as standard but was incrementally increased if there was failure of stone fragmentation. Fragments were either flushed into the duodenum (mainly in cases with previous sphincterotomy) or withdrawn with the Dormia basket via the cystic duct [18]. Choledochoscopy aimed to view the entire CBD, including the proximal ducts, to ensure complete duct clearance. If there was failure to achieve complete stone extraction or suboptimal tip deflection via the TC route (or if the cystic duct was absent), a choledochotomy was performed.

### Data analysis

Patients requiring LABEL were compared to all those not requiring LABEL. The purpose of this comparison was to assess for laser specific complications, and as such, demographic characteristics and pre-operative investigations relating to biliary disease were also compared.

IBM SPSS Statistics Grad Pack Version 24 (Chicago, IL, USA) was used for data analysis. Binary data was analysed with the chi-squared test for proportions and nonparametric continuous data was analysed with the Mann-Whitney U test (*p* of < 0.05 was considered significant).

## Results

Since the introduction of LABEL at out institution, 179 consecutive patients underwent LCBDE between February 2014 and December 2018. The median age was 56.0 (IQR 40–71) years, and 60 (33.5%) patients were male. Seventy-six (42.5%) patients were American Society of Anaesthesiologists (ASA) physical status classification 1, 71 (39.7%) were ASA 2, 20 (11.2%) were ASA 3, and for the remainder of patients (6.7%), no ASA classification was recorded. LABEL was utilised in 36 (20.1%) cases, and stone clearance was achieved in 177 (98.9%) patients. All procedures were completed laparoscopically with zero cases converted to open surgery. Within the whole series, 20 (11.2%) patients experienced post-operative morbidity, 17 (9.5%) of which were Clavien-Dindo I–II and 3 (1.7%) were Clavien-Dindo III–IV. There was no post-operative in-hospital mortality.

**Fig. 1:**
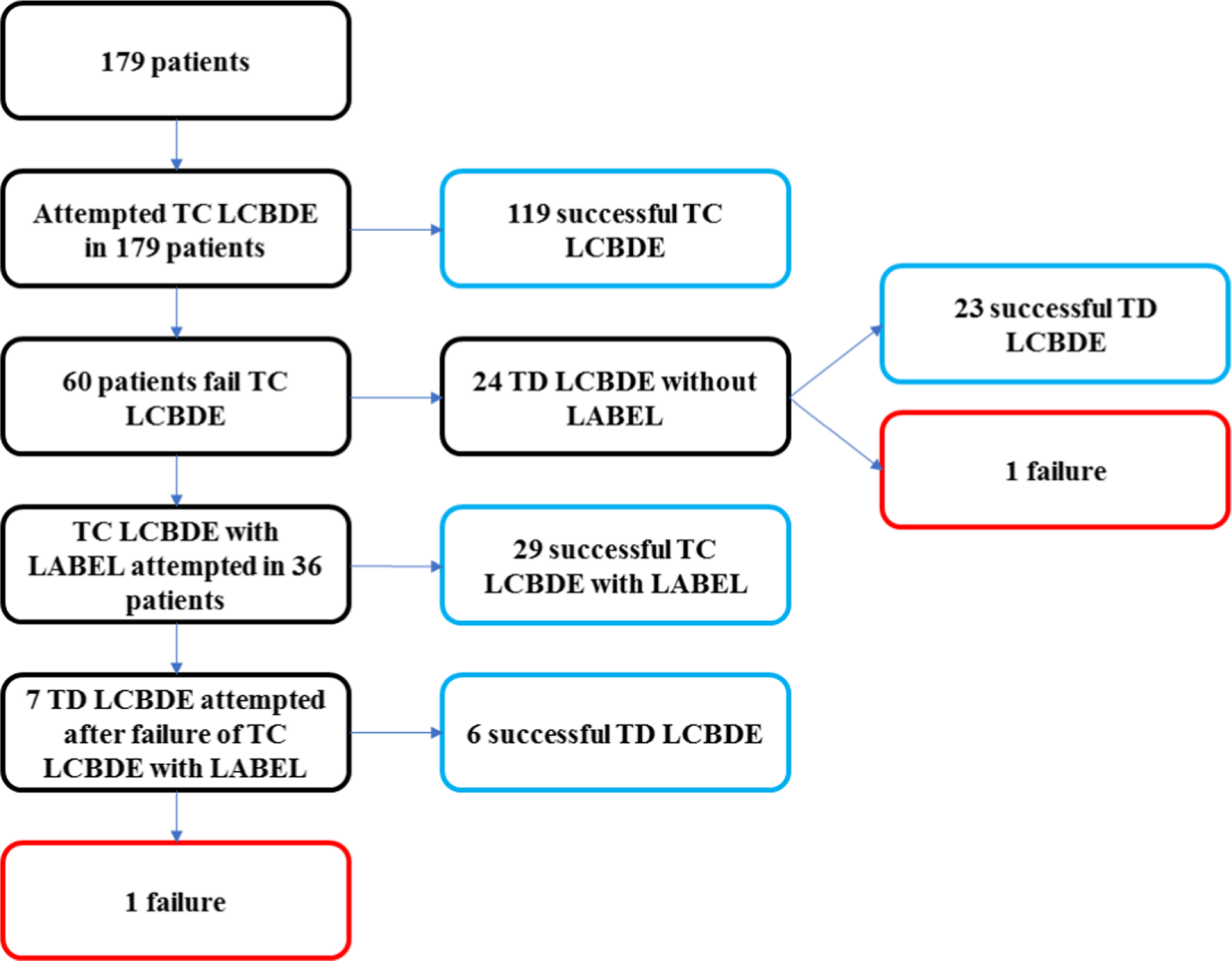
Patient flowchart representing which interventions were performed on 179 consecutive patients with choledocholithiasis. See text for full details (LCDBE, laparoscopic common bile duct exploration; TC, transcystic; TD, transductal; LABEL, laser-assisted bile duct exploration by laparoendoscopy)

TC LCBDE was planned in all 179 patients and was successfully completed in 119 of these without the need for LABEL (Fig. 1). Of the 60 patients that failed this approach initially, 36 underwent LABEL, and 24 underwent TD LCBDE immediately due to non-availability of holmium laser, a need to reduce operative and therefore total anaesthesia time in frail or multiply co-morbid patients or at the surgeon’s discretion. LABEL allowed successful TC LCBDE in 29 patients, but 7 patients required conversion to choledochotomy and TD LCBDE, of which 1 failed and the remainder were successful. Of the cohort that underwent TD LCBDE without attempting LABEL, 23 were successful and 1 failed. Overall 148 patients were successfully treated using a TC approach (of which 29 required LABEL for this), and 31 were carried out with a TD approach after TC LCBDE failed. From our previous experience before LABEL was introduced, cases failing the TC approach would have progressed directly to choledochotomy and TD stone extraction, and as such, we consider that these 29 LABEL cases were ‘saved’ from undergoing choledochotomy and TD LCBDE (Fig. 1). Therefore, within our series, LABEL theoretically increased the successful rate of TC LCBDE from 66.5% (119/179) to 82.7% (148/179). The two cases in our series that failed to achieve complete stone clearance with LCBDE were identified intra-operatively and were both treated successfully with post-operative ERCP.

When all cases utilising LABEL (n = 36) were compared to the remaining cases (n = 143), baseline demographic characteristics were similar (Table 1). The groups were comparable for pre-operative physical status and co-morbidities, with a similar rate of cardiovascular (36.1% vs 36.4%, *p* = 0.978) and respiratory disease (11.1% vs 20.3%, *p* = 0.205) and proportion of higher risk patients (ASA 3: 11.1% vs 11.2%, *p* = 0.989). However, there were significantly more patients with diabetes and other endocrine disorders in the standard group (5.6% vs 28.7%, *p* = 0.004). A greater proportion of patients undergoing the LABEL procedure presented with jaundice (47.2% vs 25.2%, *p* = 0.01), and these patients had correspondingly higher median bilirubin levels (42 [12–112] vs 19 [8–50] mmol/L, *p* = 0.014) but had similar transaminase levels (Table 1). Likewise, this group was significantly more likely to have failed pre-operative ERCP, defined as failure to cannulate or completely clear the CBD (33.3% vs 7.7%, *p* = 0.0001).

**Table 1:** Comparison of the background medical and demographic characteristics, pre-operative function and intra-operative findings in patients undergoing LABEL vs standard LCBDE (LCDBE, laparoscopic common bile duct exploration; LABEL, laser-assisted bile duct exploration by laparoendoscopy; ASA, American Society of Anaesthesiologists physical status classification; CBD, common bile duct; ERCP, endoscopic retrograde cholangiopancreatography; IQR, inter-quartile range; ALP, alkaline phosphatase; ALT, alanine aminotransferase; TC, transcystic route)

	LCBDE requiring LABEL (n = 36)	LCBDE not requiring LABEL (n = 143)	*p* value
Median age (IQR)	47 (37–70)	58 (43–71)	0.077
Gender (% male)	14 (38.9%)	46 (32.2%)	0.445
*Pre-operative fitness for surgery (%)*			
ASA 1	18 (50%)	58 (40.6%	0.306
ASA 2	10 (27.8%)	61 (42.7%)	0.103
ASA 3	4 (11.1%)	16 (11.2%)	0.989
*Medical co-morbidity (%)*			
Cardiovascular	13 (36.1%)	52 (36.4%)	0.978
Respiratory	4 (11.1%)	29 (20.3%)	0.205
Diabetes and other endocrine	2 (5.6%)	41 (28.7%)	0.004
Other	7 (19.4%)	45 (31.5%)	0.156
*Clinical presentation (%)*			
Dilated CBD	10 (27.8%)	34 (23.8%)	0.618
Deranged LFTs	7 (19.4%)	48 (33.6%)	0.101
Jaundice	17 (47.2%)	36 (25.2%)	0.01
Pancreatitis	2 (5.6%)	25 (17.5%)	0.074
Previous unsuccessful ERCP	12 (33.3%)	11 (7.7%)	0.0001
*Pre-operative LFTs*			
Median Bilirubin, mmol/l (IQR)	42 (12–112)	19 (8–50)	0.014
Median ALP, U/L (IQR)	182 (111–321)	186 (108–283)	0.367
Median ALT, U/L (IQR)	166 (51–333)	116 (48–302)	0.581
*Diameter of CBD on pre-operative imaging*			
Median CBD diameter, mm (IQR)	12 (10–15)	10 (8–12)	0.0001
*Intra-operative data*			
TC LCBDE (%)	29 (80.6%)	119 (83.2%)	0.784
Median number of stones (IQR)	2 (1–3)	1 (0–3)	0.007
Median size of largest stone (IQR)	10 (7–15)	5 (4–7)	0.0001
Median operative time, min (IQR)	135 (115–175)	112 (90–145)	0.001

Patients requiring LABEL group appeared to have more complex gallstone pathology when compared with those who did not require LABEL, with more ductal calculi (2 [1–3] vs 1 [0–3], *p* = 0.007), greater size of the largest stone (10 [7–15] vs 5 [4–7] mm, *p* = 0.0001) and larger CBD diameter (12 [10–15] vs 10 [8–12] mm, *p* = 0.0001). As expected, operative times within the LABEL group were significantly longer (135 [115–175] vs 112 [90–145] minutes, *p* = 0.0001). The LABEL group had similar rates of stone clearance (Table 2) when compared to the standard group (97.2% vs 99.3%, *p* = 0.248). Patients requiring LABEL also had longer post-operative stay (2 [1–6] vs 1 [1–3] days, *p* = 0.022).

**Table 2:** Comparison of operative outcomes and complications in patients undergoing LABEL vs standard LCBDE (LCDBE, laparoscopic common bile duct exploration; LABEL, laser-assisted bile duct exploration by laparoendoscopy; IQR, inter-quartile range)

	LCBDE requiring LABEL (n = 36)	LCBDE not requiring LABEL (n = 143)	*p* value
Stone clearance (%)	35 (97.2%)	142 (99.3%)	0.248
Conversion to open surgery (%)	0	0	
Median length of post-operative stay, days (IQR)	2 (1–6)	1 (1–3)	0.022
*Complications (%)*			
Clavien-Dindo I–II	6 (16.7%)	11 (7.7%)	0.101
Medical complications	4 (11.1%)	7 (4.9%)	0.165
Pancreatitis (mild)	0	2 (1/4%)	0.476
Mild bile leaks	0	2 (1.4%)	0.476
GI bleed (conservative management)	1 (2.8%)	0	0.046
Liver haematoma (conservative management)	1 (2.8%)	0	0.046
Clavien-Dindo III–IV	2 (5.6%)	1 (0.7%)	0.042
Major bile leak (requiring re-intervention)	2 (5.6%)	0	0.005
Acute pancreatitis (severe)	0	1 (0.7%)	0.615
Clavien-Dindo V (30-day mortality)	0	0	

The overall rate of minor complications was similar between the two groups (Clavien-Dindo I–II: 16.7% vs 7.7%, *p* = 0.101) although one patient in the LABEL group suffered from a mild GI bleed that was managed conservatively and another developed a small liver haematoma, also managed conservatively (Table 2). However, there were significantly more major complications within the LABEL group (Clavien-Dindo III–IV: 5.6% vs 0.7%, *p* = 0.042). None of these complications were specifically attributable to the laser intra-operatively. Instead, they were due to two major bile leaks requiring re-intervention post-operatively (6.5% vs 0%, *p* = 0.005). One patient had a bile leak from the duct of Luschka and re-presented after discharge with peritonitis, requiring exploratory laparotomy and washout. The other patient had a bile leak from the CBD and underwent treatment with post-operative ERCP.

## Discussion

Our results demonstrate that LABEL is an effective adjunct to LCBDE and is able to increase the number of cases successfully performed via the transcystic route. Approximately one fifth (36/179) of patients required LABEL, and these cases were more complex with a greater number and size of ductal calculi with correspondingly higher rates of failed pre-operative ERCP. However, the LABEL group had more bile leaks requiring intervention, longer operative times and longer post-operative hospital stay. These results may well be related to the greater complexity of cases within the LABEL group rather than related to laser lithotripsy itself.

LCBDE, as part of a single-staged procedure, is now emerging as the preferred treatment option for patients with choledocholithiasis and gallbladder in situ, provided that the necessary expertise are available [1]. ERCP has traditionally formed the mainstay of therapy for CBD stones, with guidelines recommending that it is best performed around the time of the index laparoscopic cholecystectomy [5]. Furthermore, there have also been recent developments in ERCP technology, including the use of laser lithotripsy, that have further increased its utility, and some centres depend almost entirely on ERCP for CBD stone removal [19, 21]. However, with increasing financial pressures on health services worldwide, therapies that facilitate a shorter hospital stay and lower overall cost will likely be the future model. Single-staged LCBDE has consistently been shown to be more cost-effective and at least as, if not more, effective as a two-staged approach [1, 22–25].

Several studies have reported the benefits of TC over TD LCBDE [6, 7, 25, 26]. Although CBD clearance rates are similar between TC and TD approaches, TC LCBDE has lower overall complications, biliary complications, bloods loss and reduced length of hospital stay. Adjuncts that improve the rate of TC LCBDE are therefore critical to enhancing the success of LCBDE as a whole especially when compared to staged procedures with ERCP. This is especially true in circumstances that are not traditionally amenable to TC stone extraction, such as impacted and/or large stones, distal stones and anatomical defects [27].

The use of laser lithotripsy as an adjunct to LCBDE was first described in a small five-patient case series, which demonstrated efficacy even when faced with large and/or impacted ductal calculi [13, 17]. Subsequently, larger studies have reported its safety and efficacy, demonstrating no associated increase in biliary related or other complications [15, 16, 28, 29]. Results from this study have comparable rates of duct clearance to these larger series but suggest that there is a small but significant increase in the rate of post-operative bile leak with LABEL cases. However, there are major methodological differences between these studies and the study we present here, making direct comparison difficult. One study excluded patients if the transcystic route was not possible [28], and another study excluded patients presenting acutely with cholecystitis and obstructive jaundice or having failed ERCP [16]. These factors most likely decrease the complexity of reported cases in previous cohorts compared to the present study, which included patients with acute presentations and those requiring TD LCBDE. Furthermore, these studies have not directly compared LABEL with standard cases but instead report complications for the cohort as a whole. A recent meta-analysis has suggested that the use of the holmium laser in LCBDE not only increases successful duct clearance but may even be associated with a shorter operative time and a shorter post-operative stay when compared to standard retrieval [30]. Our results are likely to be discrepant to these as our institutional practice only selected patients for LABEL that had failed standard retrieval techniques and therefore had more complex biliary pathology.

To our knowledge, this is the first Western study to highlight the utility of LABEL to increase the ability to perform TC LCBDE; our centre is now able to perform the vast majority of operations via this route. Xia et al. report a Chinese cohort with a similar increase (63.5% to 93.7%), also assuming that cases requiring laser would have required TD LCBDE had it not been available [16]. Both results suggest that without access to the holmium laser, even in experienced hands, the proportion of TC LCBDE is likely to be around two thirds at the most. As such, surgeons pursuing training in LCBDE will be increasingly required to become familiar with laser technology and safety.

Predicting when LABEL may be required as part of LCBDE would be useful in ensuring that the correct personnel and equipment are available. Our results suggest that patients presenting with obstructive jaundice (and with higher bilirubin levels) or having failed pre-operative ERCP are significantly more likely to require LABEL. Interestingly neither transaminase levels nor CBD diameter on pre-operative imaging was associated but number and maximum size of ductal calculi were. As such, we suggest that appropriate pre-operative work up to establish this information (including potentially MRCP) can assist in procedure planning, ensuring the availability of both the laser itself and of the trained auxiliary staff required for its safe and effective application.

Another procedure that has recently gained traction is the so-called laparoscopic-endoscopic rendezvous procedure, which involves ERCP at the same time as LC [31, 32]. It has shown benefit in reducing the length of hospital stay over the conventional two-staged procedure, although a recent meta-analysis concluded that there was insufficient evidence to recommend its utility [33]. We note that almost one third of patients requiring LABEL for successful clearance in our cohort had failed ERCP pre-operatively, including one of the patients experiencing a major bile leak. In our experience, these findings cement LABEL as an adjuvant technique to LCBDE that is effective even when ERCP has failed.

The key limitations of this study relate to its retrospective, non-randomised nature where patients allocated to the LABEL group were, by definition, those who had failed standard retrieval. These factors introduce significant allocation bias which decreases the comparability of the two groups, although notably both groups had similar baseline demographic and medical characteristics. Retrospectively analysing data can also lead to reporting bias. However, given the results were mostly based on binary outcomes such as success and complication rate, this is likely to be minimised in our study. Furthermore, the study was not designed, or indeed powered, to make comparisons between patients undergoing TC LCBDE with LABEL and TD LCBDE without LABEL. Instead it aimed to demonstrate that the technique was safe and effective at improving the rate of TC stone extraction. It is presumable that if a larger cohort was studied, the benefits of increased TC access would become apparent in those undergoing this technique. Further studies, with larger patient numbers and that randomise patients that initially fail TC LCBDE to either TD LCBDE or TC LCBDE with LABEL or intra-operative ERCP, are needed to conclusively prove its benefit in terms of endpoints like length of hospital stay and biliary morbidity. A final limitation is that cost-effectiveness data was not available. Future studies should consider whether laser technology is a worthwhile investment for biliary surgery; it may be most cost-effective in larger volume centres such as our own, where over one fifth of cases utilised LABEL or by sharing the equipment with other surgical subspecialties such as Urology.

In terms of future directions for laser lithotripsy in biliary disease, there have been numerous studies trialling a novel laser system, the FREDDY (frequency-doubled double-pulse neodymium: YAG) laser as part of ERCP [34, 35]. This solid-state laser has been developed specifically for in vivo usage against biliary and ureteric stones and utilises short pulses of dual frequency energy applied simultaneously [36]. About 20% of its emission is in the green spectrum (532 nm) and initiates plasma formation on the surface of the stone, whilst 80% is in the infrared spectrum (1064 nm) and causes repeated expansion and shrinkage of this plasma layer, resulting in mechanical, rather than thermal, destruction [37]. In vitro studies have demonstrated minimal tissue damage with the FREDDY laser, even upon direct application to animal or human epithelial tissue, especially when compared to the standard holmium laser [38, 39]. More recently, this system has been applied via TC LCBDE with excellent results, and it is likely that its use will become more widespread in the future [28, 29]. Furthermore, the use of EHL has recently gained popularity due to the fact that it utilises high-voltage electric sparks, rather than laser radiation, and hence requires less specialised theatre equipment [8]. Its use has mainly been limited to ERCP, however, partly due to the success of laser lithotripsy as a surgical technique [12].

## Conclusion

Our study suggests that LABEL is an effective adjunct to LCBDE that can increase the rate of successful transcystic approach but is associated with an increased risk of major complications, though none specifically attributable to the laser itself. It can be utilised for the most complex cases, where standard retrieval has failed, with excellent results. Future research should focus on maximising the efficacy and safety of LABEL by determining the optimum laser system and operative technique.
